# EAM highlights in FEMS 2023: from the Petri dish to planet Earth

**DOI:** 10.1093/femsml/uqad045

**Published:** 2023-11-03

**Authors:** Jörg Vogel, Victor de Lorenzo

**Affiliations:** Helmholtz Center for Infection Research, Helmholtz Institute for RNA-based Infection Research and Institute for Molecular Infection Biology, University of Würzburg, Würzburg, Germany; Systems Biology Department, National Center of Biotechnology CSIC, Madrid, Spain

**Keywords:** phage, giant virus, antibiotic, antimicrobial resistance, microbiome, environmental microbiology

## Abstract

On 9–13 July 2023, the 10th FEMS Congress took place in Hamburg, Germany. As part of this major event in European microbiology, the European Academy of Microbiology (EAM) organized two full sessions. One of these sessions aimed to highlight the research of four recently elected EAM fellows and saw presentations on bacterial group behaviours and development of resistance to antibiotics, as well as on new RNA viruses including bacteriophages and giant viruses of amoebae. The other session included five frontline environmental microbiologists who showcased real-world examples of how human activities have disrupted the balance in microbial ecosystems, not just to assess the current situation but also to explore fresh approaches for coping with external disturbances. Both sessions were very well attended, and no doubt helped to gain the EAM and its fellows more visibility.

The European Academy of Microbiology (EAM) was founded by the Federation of European Microbiological Societies (FEMS) and consists of a distinguished panel of esteemed experts in the field of microbiology. One major element of the EAM’s mission is to enhance the visibility and prominence of microbiology itself, as well as of the leading European researchers in this field. This overarching goal is centred on advancing excellence in microbiology across both the European landscape and the global stage. To fulfil this mission, the EAM employs targeted programms and initiatives designed to push the boundaries of the discipline, while also ensuring effective communication of these endeavours to scientists, stakeholders, and the public.

At the 10th FEMS Congress of European Microbiologists (FEMS2023), the EAM organized and held two sessions, one organized and chaired by EAM President Jörg Vogel (Würzburg, Germany), the other by EAM Executive Board member Victor de Lorenzo (Madrid, Spain).

## Session with highlights from the EAM

**Figure 1.**
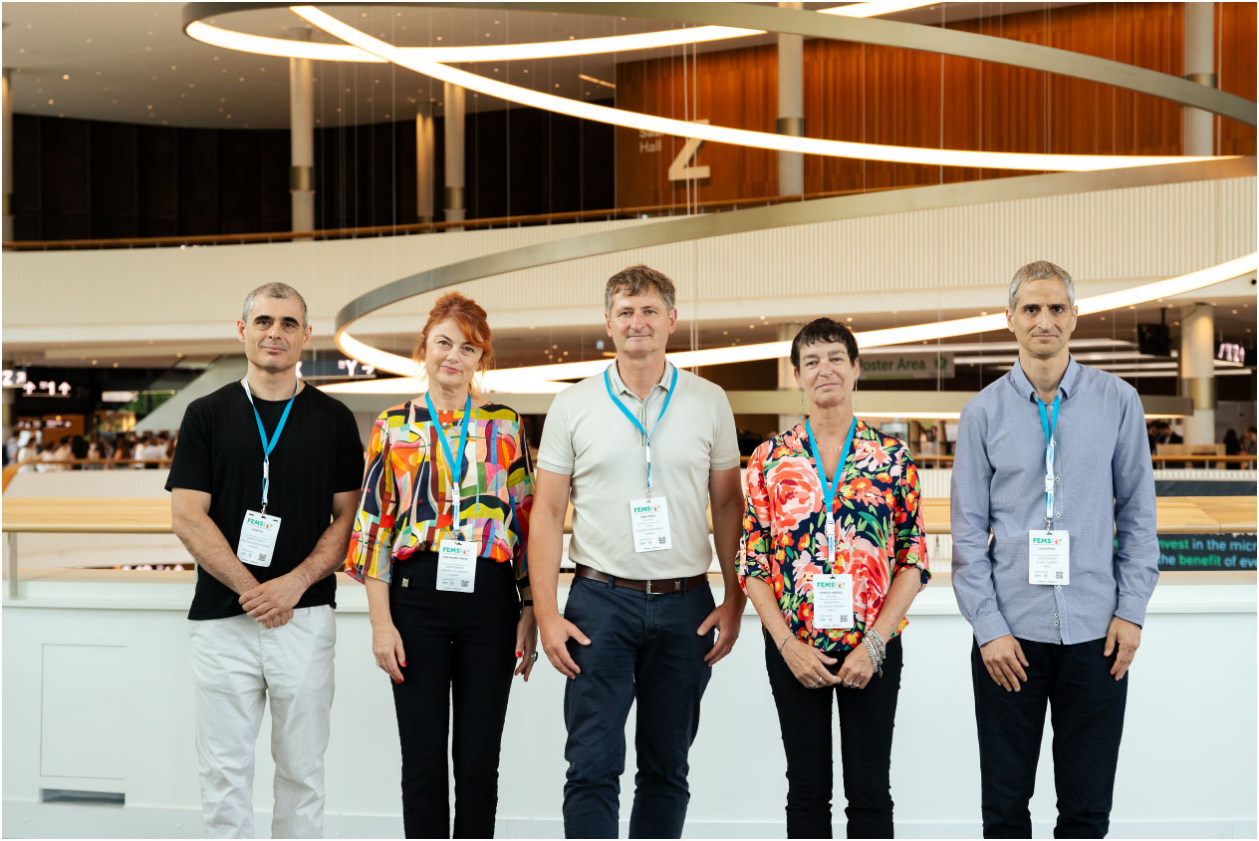
From left to right: Csaba Pal, Ines Mandic-Mulec, Jörg Vogel, Chantal Abergel, and Uri Gophna.

The EAM currently is 160 fellows strong. New fellows are elected every other year, through a structured process that involves external nomination and a vote by all fellows. The purpose of the first session, 2 hours long, was to highlight the breadth of research of recently elected fellows. To this end, EAM President Jörg Vogel, invited fellows from four different countries to present an overview of their recent and ongoing research, that is, Ines Mandic-Mulec (Slovenia, elected 2022), Uri Gophna (Israel, 2017), Chantal Abergel (France, 2017), and Csaba Pal (Hungary, 2017) (Fig. 1).

The session was kicked off by Ines Mandic-Mulec, who is a Professor of Microbiology and currently serves as the Vice-dean of the Department of Microbiology at the Biotechnical Faculty of the University of Ljubljana, Slovenia. Her laboratory has had a strong interest in molecular mechanisms, consequences, and evolution of bacterial group behaviours. In her presentation, she focused on kinship-dependent social behaviours as observed with the model soil-dwelling bacterium, *Bacillus subtilis*, which nicely illustrated that and how microbes can be social organisms. One of her experimental approaches is to look for kinship-dependent differential behaviours within the same species, using bacteria that were isolated from soil patches only a few meters apart. It was fascinating to see examples of very different swarming behaviour: closely related swarms merge, in contrast, less related swarms form a boundary at which killing occurs (Stefanic et al. 2015, Kraigher et al. 2022). She also presented recent results to suggest that swarm combat promotes horizontal gene transfer with a potential involvement of these bacteria’s ability to respond to envelope stress (Stefanic et al. 2021). Lastly, she discussed the role of so-called exploiters and whether kin discrimination occurs because it limits the spread of exploiters in evolved populations.

The second talk of the session was given by Uri Gophna, who is a Professor of Microbiology at the Faculty of Life Science, Tel Aviv University, Israel. While he broadly researches into microorganisms from all domains of life, he has recently made a big splash in the discovery of viruses of bacteria and archaea. In Hamburg, he presented two recent stories, the first of which is based on a large metatranscriptome study that has led to a multifold expansion of the microbial RNA virosphere (Neri 2022). Compared with their DNA counterparts, RNA viruses are much less understood with respect to their diversity and role in microbial ecosystems, which is partly due to a discovery bias. Mining several thousands of metatranscriptomes from various environments, an international collaboration led by Gophna established an extensive catalogue of RNA virus genomes. This catalogue produced two new phyla, including one with new RNA phages. The many newly predicted genes present not only exciting opportunities to better understand virus–host interactions but also potential new molecular tools for biotechnology. In his other story, yet unpublished, he showed fascinating observations with a lemon-shaped archaeal virus that infects *Haloferax* species and influences mating behaviour. Using RNA-seq, the laboratory is seeking to leverage transcriptome-derived information to understand how this virus alters the behaviour of virus-infected strains.

**Figure 2.**
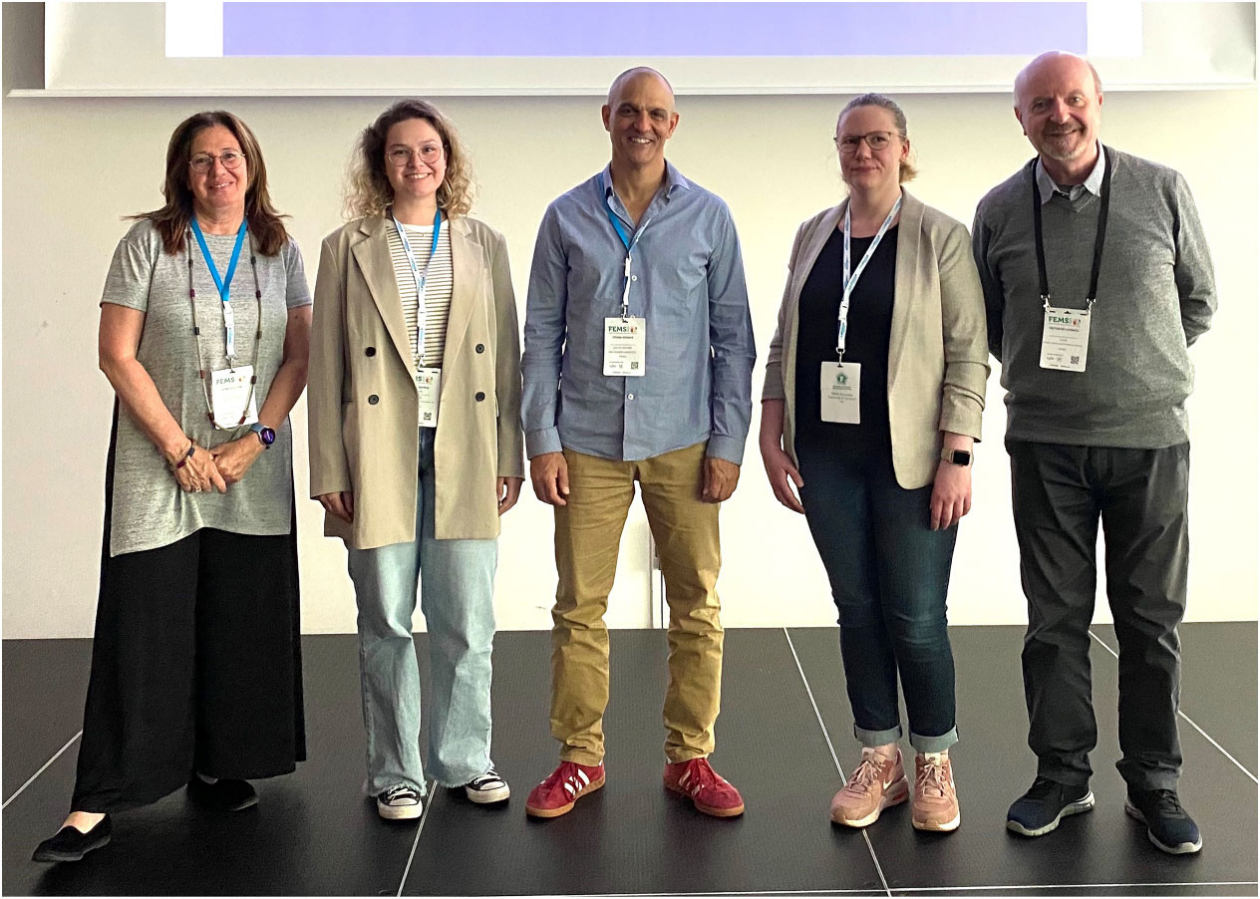
From left to right: Laura Zucconi, Rebecca Bärhle, Itzhak Mizrahi, Nicole Adam-Beyer, and Victor de Lorenzo.

The third talk of the session continued on the theme of virus and was given by Chantal Abergel, who is a cofounder of the Structural and Genomic Information (IGS) laboratory at CNRS Marseille, France. Her laboratory combines bioinformatics with experimental biology. Here, she spoke about genome organization in giant viruses infecting Acanthamoeba. Starting with the original discovery of the giant mimivirus, whose genome turned out to be three times the size of the known large *Chlorella* virus (Raoult 2004), she showed how the isolation of many more environmental DNA viruses later produced more of a continuum such that there are many eukaryotic viruses that possess genomes several hundreds of kilobases and up to > 2.8 megabase in size. The mimivirus virion is more like a Russian doll, with particles of highly complex structure in which genomic fibre is interspersed with molecules of RNA polymerase; an elegant supramolecular organization that ensures that the genome can be safely packaged but viral transcription can jump-start upon unwinding of these genomic fibres in the host cytoplasm (Fig. 2). This fine-grained picture has emerged from the integration of diverse high-resolution structural data, running the gamut from traditional X-ray crystallography to cryogenic electron microscopy (cryoEM) and electron cryotomography (cryoET) (Villalta 2022). Puzzling observations remain, for example the GMC oxidoreductase composing the genomic fibre is not essential its formation. Abergel concluded her talk by asking the provocative question of why giant viruses such as the mimiviruses evolved in the first place. Is their gigantism just a consequence of an increasing functional redundancy in the viral world?

The session concluded with a talk by Csaba Pal, whose laboratory belongs to the Synthetic and Systems Biology Unit at the Biological Research Centre in Szeged, Hungary. He asked key questions about the mechanisms and evolutionary path of resistance development for future antibiotics that are just being developed. This is not a small question, as it takes a company > 20 years to make a profit from a new antibiotic. If resistance develops early after the start of use in the clinics, this will kill the overall investment, which can easily be in the range of a billion US dollars. Because studying antimicrobial resistance in the lab is a complex task, Pal has built a powerful pipeline to assess resistance development (Apjok et al. 2023, Nyerges et al. 2018, Spohn et al. 2019), with a focus on Gram-negative bacteria such as the concerning bugs *Escherichia coli, Klebsiella* spp., *Pseudomonas aeruginosa*, and *Acinetobacter baumannii*. Using his pipeline, he has discovered a substantial overlap in the molecular mechanisms of resistance against clinically used and new antibiotics. These data will be important to come up with new combinations of antibiotics that will continue to work in the future and argue for more narrow-spectrum therapy. He concluded his presentation by showing data on how resistance might change bacterial virulence, using the example of SPR206, a promising peptide antibiotics. Alarmingly, it appears that SPR206-resistant *Klebsiella* bacteria, which emerge rapidly after treatment, are also much more virulent, presumably due to changes in their envelope stress pathway. Thus, resistance may select for hypervirulent bacteria, a situation that must be avoided in the clinics.

## EAM symposium: microbial responses to environmental threats

The second scientific event organized by EAM was the Symposium entitled *Microbial Responses to Environmental Threats*, with chair Victor de Lorenzo and speakers from four different countries (Fig. 2). The rationale and motivation behind the sequence of talks of this session were the presentation of several exemplary instances where anthropogenic activities have had a serious impact on the homeostasis of existing microbial ecosystems—whether marine, terrestrial, or animal-associated. To this end, the expectation was not only a mere stocktaking of the state of affairs, but also gathering some insights on the metrics and technologies for monitoring microbial communities, their composition and their variations upon external perturbations. Furthermore, the issue at stake was not only engaging in descriptions of the current picture (grim as they may look at the moment) but also entertaining forward-looking prescriptions fuelled by contemporary Systems and Synthetic Biology.

The Symposium was opened by short talks of two early career scientists of the GEOMAR-Helmholtz Center for Ocean Research in Kiel, Germany. The first of them, Nicole Adam-Beyer, discussed the dynamics of microbial communities under seasonal hypoxia. This is an important issue, as human activities are modifying the oceanic environment rapidly and are causing ocean warming and deoxygenation, affecting biodiversity, productivity, and biogeochemical cycling. She presented studies on the seasonally dynamic Boknis Eck time series station (SW Baltic Sea), where bottom waters annually fall hypoxic or anoxic after the summer months. Geochemical and microbiological (16S rRNA-based) analyses of sediments were used to extrapolate how the benthic microbial communities and the associated metabolic activities reflect rising temperatures and deoxygenation. Based on these data, the localization of the microbial populations catalyzing sulphide and methane metabolisms seems to change to shallower sediment horizons following hypoxic events and a shift from a primarily organotrophic to an (autotrophic) sulphide oxidizing regime was observed (Perner et al. 2022). Continuous sampling campaigns will be used to further characterize the effects of hypoxic events and enhanced microbial sulphide production in the Baltic Sea.

The second GEOMAR speaker, Rebecca Bährle, explained ongoing strategies for finding new carbon monoxide dehydrogenases (CODHs). These are superinteresting enzymes as the catalyze the reaction between carbon monoxide (CO) with water to carbon dioxide (CO_2_) protons and two electrons—and therefore, these enzymes can help to convert the greenhouse gas CO_2_ into valuable commodities. Bährle shared her strategies for detecting such activity in metagenomic libraries by means of a colorimetric screening approach that detects oxidation of CO to CO_2_. Anoxic marine sediments appear to be a good hunting ground of bacteria carrying new catalysts of this sort. An interesting debate followed on how such improved enzymes—once identified, cloned, and so on—could be delivered at large scale in either an industrial setting or a large environmental scenario.

These two starting presentations were followed by an amazing talk by Laura Zucconi from the University of Tuscia, Viterbo, Italy, on her long-time monitoring of soil communities in polar and alpine ecosystems hit by global warming. Remarkably, such environments are among the most endangered from climate change, making them hotspots for studies on the effects on soil ecosystems. Although heat-induced upward plant migration has been well-documented in high altitude regions, the responses of the associated soil microbial communities has been overlooked. Of considerable interest is the observation that at lower altitude, shrubland had the highest proportion of fungi, which was correlated with higher amounts of enzymes for degrading biomass and recalcitrant plant biopolymers. Shrub encroachment may accelerate higher recalcitrant C decomposition and reduce total ecosystem C storage, increasing the efflux of CO_2_ to the atmosphere with a positive feedback to warming. However different studies in different environments are providing different pictures, therefore multidisciplinary studies, integrating different warming-simulation approaches, are needed to give a clear picture of the responses of these ecosystems (D’Alò et al. 2021, 2022). Instead, in Antarctica, the prediction is that climate change will lead to extinction of unique highly adapted taxa. The strong correlation of the communities’ diversity and composition with soil abiotic properties is of particular concern in the light of possible shifts of environmental conditions (Severgnini et al. 2021).

The issue of superfunctionalities of microbial communities initiated by Laura was a perfect prologue of the presentation by Itzhak Mizrahi from the Ben Gurion University, Bersheeva, Israel. In an entirely different context, the subject in this case was unveiling the microbiome–metabolome interplay in a system as extremely complex as the rumen a distinct compartment in the bovine digestive tract. The rumen microbiome is responsible for the production of one of the most potent greenhouse gases, methane, and contributes about 18% of its total anthropogenic emissions. In this case, unlike soil and marine ecosystems, the physical container of the community is itself a contained, living scenario with its own properties and dynamics. This adds one more screw turn to the technical challenge of solving metabolic interactions among all partners not just in terms of composition and biochemical transactions but also as influenced by the 3D organization of the bacterial consortia involved. In this respect, the link between taxonomy and cellular/community architecture, has been recently tackled with cryoelectron microscopy and tomography, enabling to decipher microbial interactions and phenotypes at the nanoscale resolution (Tatli et al. 2022). These methods promise to become a phenomenal tool for addressing similar questions in other complex microbiomes.

Finally, to transition the Symposium’s focus from description to prescription, Víctor de Lorenzo concluded the session by sharing his recent endeavours in the National Center of Biotechnology CSIC in Madrid. He highlighted ongoing efforts to harness the latest advancements in strain and community engineering, made possible by Synthetic Biology, for the purpose of extensive environmental bioremediation. He delved into the exploration of an optimal bacterial chassis, which could serve as the preferred platform for crafting designer live agents (de Lorenzo 2022). These agents would effectively and safely execute synthetically primed environmental activities on a large scale. As an example, he elaborated on the concept of ectopically expressing camel antibodies (nanobodies) on the surface of environmental bacteria, such as *Pseudomonas putida*. This approach facilitates the formation of microbial skins on solid surfaces (Fraile et al. 2021) and the creation of more proficient catalytic communities. However, realizing these approaches at a large scale necessitates addressing numerous knowledge gaps, developing robust dispersion models, and initiating a renewed societal and regulatory discourse regarding the controlled release of engineered catalysts into the environment.

## Conclusion

Naturally, each presentation of either Symposia was succeeded by a lively discussion among the participants, poised to yield fresh collaborations and experiments. In essence, each of the two EAM events served as a testament to some of the most interesting scientific questions of our time as well as a display of the top concerns of our era, while providing compelling evidence regarding the role and potential of microorganisms. These function as sensitive indicators of environmental shifts while also serving—by themselves of with some rational priming—as agents capable of dealing with the impacts of human activities on global health.
